# Editorial: Emerging talents in toxicology

**DOI:** 10.3389/ftox.2024.1369297

**Published:** 2024-02-01

**Authors:** Takamitsu A. Kato

**Affiliations:** Department of Environmental and Radiological Health Sciences, Colorado State University, Fort Collins, CO, United States

**Keywords:** nanoparticle, Corona formation, model organisms, risk assessment, nanodebris

In this editorial, I summarize six articles associated with nanoparticle research in the Research Topic “Emerging Talents in Toxicology” of the Journal of Frontiers in Toxicology.

The Research Topic, titled “Emerging talents in Toxicology” highlights recent advancements in toxicology, not limited to nanoparticle toxicology. The Research Topic comprises nine papers, with six of them specifically addressing nanoparticles. The growing use of nanomaterials indeed brings both benefits and concerns. Nanomaterials, due to their unique properties, have applications in various fields such as medicine, electronics, and materials science. However, the potential exposure to nanodebris raises questions about their impact on human health and the environment. It is crucial to distinguish between intentionally produced (engineered) nanomaterials and nonodebris. The term of ‘nanodebris’ refers to nanoparticles originating from the decomposition and wear-off materials a Research Topic specifically addressed in nanoplastics by Cunningham et al. This distinction is essential for a comprehensive understanding of the implications associated with nanomaterial usage. The specific risks associated with nanomaterial exposure depend on factors like the types of nanomaterial, its size, shape, and the route of exposure. Research is ongoing to understand the potential health and environmental effects of nanomaterials. In the context of human exposure, concerns include inhalation of airborne nanoparticles, ingestion through contaminated food or water, and skin contact. Similarly, environmental exposure can occur through the release of nanomaterials into air, water, or soil. Although nanoparticle research is expanding rapidly, there remain many un-answered questions in the fields. The six articles contribute insights into various nanoparticles, including Silver nanoparticle, Titanium dioxide nanoparticle, Cadmium Tellurium Quantum dots, and nanoplastics.

This editorial briefly introduces these papers, with three research papers investigating the detection and toxicology of nanoparticle and protein interactions. Another research article explores the transgenerational effects of nanoparticle exposure. Two review papers are included, with one summarizing the current status and problems of nanoplastics pollution and risk assessment, which the other proposes a unique model organism for ecotoxicology research.

The study by Park et al. investigates *in vitro* nanoparticle toxicology with protein modification, specifically examining the impact of glycation on nanoparticle-protein interactions, known as Corona formation. Silver nanoparticles, wildly used in the medical field for their antimicrobial properties, were studied in relation to their interaction with Human serum albumin (HSA) in human liver carcinoma HepG2 cells. The study found that HAS interaction increased the toxicity of silver nanoparticles to HepG2 cells without dissolving silver ions, suggesting potential novel properties in Corona formation. In the study by Griffith et al., *in vivo* nanoparticle toxicology with nano-Titanium dioxide is explored through an inhalation study using pregnant Sprague-Dawley rats. The study hypothesizes a sex-dependent toxic effect of nanoparticles on the fetus through the placenta. Maternal inhalation exposure to nano-Titanium dioxide was found to have a greater effect on fetal females, potentially influencing their growth and development in a sex-dependent manner. Bosch et al. investigated *in vivo* toxicity of cadmium tellurium quantum dot nanomaterials with different functional groups (carboxylate, ammonia, polyethylene glycol) in zebrafish embryos. Quantum dots, fluorescent nanocrystals with a semiconductor metal core, are used as contrasting agents for bio-imaging applications. The study showed that ammonia functionalized cadmium tellurium quantum dot nanoparticles caused the most severe effects, including respiration inhibition and developmental defects. Cunningham et al. provided a review highlighting critical gaps between nanoplastics research and risk assessment. Plastic pollution is a global issue, with research focusing more on macroplatics than nanoplastics due to detection challenges. The limited knowledge about the introduction and transport of nanoplastics in the environment suggests a potential underestimation of nanoplastics pollution. The authors emphasize the need for improved ecological risk assessment for nanoplastics. Reilly et al. reviewed and proposed Daphnia as a model organism to study biological responses to nanomaterials. Daphnia has been established as a model organism for ecotoxicity testing, and recent advancements such as the completion of the Daphnia genome project, enable molecular ecotoxicology approaches to understand human health implications through conserved biochemical pathways Daphnia, a legacy model organism, combined with innovative approach such as microfluidics, allows for real time analysis of nanomaterial toxicity. Umezawa et al. investigated the secondary structure change in albumin with cerium oxide nanoparticles using an IR spectroscopy-based method. Cerium oxide is crucial industrial materials with unique anti-oxide activities with cytotoxicity. The study focuses on detecting protein structure changes potentially associated with nanoparticle toxicity, which may alter cellular interaction.

It is interesting to note that the theme of “Emerging Talents in Toxicology” implies that the first authors of the articles are likely scholars in training, showcasing the involvement of young researchers in the field of toxicology. The diversity of nanoparticles and Research Topic covered in the six articles suggests a broad range of research areas within nanoparticle studies. This diversity not only reflects the expanding scope of nanoparticle research but also indicates that there are ample opportunities for further exploration and advancement in this field. As emerging talents delve into various aspects of toxicology related to nanoparticles, they contribute to the overall understanding of the potential risks and benefits associated with nanomaterials. Nanoparticle research is interdisciplinary, involving fields such as toxicology, materials science, and environmental science. The collaboration of emerging talents across these disciplines can lead to innovative approaches and insights. It is crucial to support and encourage young researchers in this area to foster continued growth and progress in nanoparticle research.

